# Memory deficits for facial identity in patients with amnestic mild cognitive impairment (MCI)

**DOI:** 10.1371/journal.pone.0195693

**Published:** 2018-04-19

**Authors:** Egemen Savaskan, Daniel Summermatter, Clemens Schroeder, Hartmut Schächinger

**Affiliations:** 1 University Hospital of Psychiatry, Department for Geriatric Psychiatry, Zürich, Switzerland; 2 Division of Clinical Psychophysiology, Institute of Psychobiology, University of Trier, Trier, Germany; University of California, San Francisco, UNITED STATES

## Abstract

Faces are among the most relevant social stimuli revealing an encounter’s identity and actual emotional state. Deficits in facial recognition may be an early sign of cognitive decline leading to social deficits. The main objective of the present study is to investigate if individuals with amnestic mild cognitive impairment show recognition deficits in facial identity. Thirty-seven individuals with amnestic mild cognitive impairment, multiple-domain (15 female; age: 75±8 yrs.) and forty-one healthy volunteers (24 female; age 71±6 yrs.) participated. All participants completed a human portrait memory test presenting unfamiliar faces with happy and angry emotional expressions. Five and thirty minutes later, old and new neutral faces were presented, and discrimination sensitivity (d’) and response bias (C) were assessed as signal detection parameters of cued facial identity recognition. Memory performance was lower in amnestic mild cognitive impairment as compared to control subjects, mainly because of an altered response bias towards an increased false alarm rate (favoring false OLD ascription of NEW items). In both groups, memory performance declined between the early and later testing session, and was always better for acquired happy than angry faces. Facial identity memory is impaired in patients with amnestic mild cognitive impairment. Liberalization of the response bias may reflect a socially motivated compensatory mechanism maintaining an almost identical recognition hit rate of OLD faces in individuals with amnestic mild cognitive impairment.

## Introduction

Mild cognitive impairment (MCI) can represent a prodromal stage to dementia, and is characterized by objective impairment in cognition with preservation of independence in functional abilities [1–4]. The prevalence of MCI is 10–20% in adults aged 65 years and older. MCI is heterogenous in its clinical presentation and the principal cognitive impairment can be amnestic, single nonmemory domain or involving multiple cognitive domains [4]. Especially the amnestic MCI (aMCI) is of interest, because individuals with this subtype may progress to Alzheimer’s disease (AD) at a rate of 10–15% per year, whereas healthy control subjects (CS) convert at a rate of 1–2% per year [5, 6]. Therefore, it is important to establish early diagnostic criteria to point out those MCI cases that may evolve into AD [7].

Cognitive problems in MCI include difficulties in memory, language, attention, and in visuospatial and executive functions which have important implications for patients and their families [2]. The key criteria that distinguish MCI from dementia are preservation of independence in functional abilities and lack of significant impairment in social or occupational functioning [2, 3]. Neuropsychological assessment and neuroimaging are screening instruments routinely used to quantify the degree of cognitive impairment. Sensitive neuropsychological tests may enhance the diagnostic quality and point out particular cognitive deficits.

There is some evidence that socio-emotional memory processing in aMCI can be impaired prior to the marked cognitive changes in dementia and those diminished abilities for emotion discrimination in aMCI may represent a prodromal phase of neuropsychological deficits in AD [8–11]. For example difficulties in recognizing anger is evident in the aMCI subytpe and impaired emotion recognition abilities impact MCI subtypes differentially [8]. Patients with AD, on the other hand, are impaired in the recognition of almost all emotional stimuli of all intensities [9, 11]. Social deficits in neurodegenerative diseases may be a consequence of emotional memory difficulties and are strongly associated with impaired behavioural functioning and care giver burden. Interestingly, impaired emotion recognition was identified only in the amnestic subtype independently of patients’ mood and other cognitive deficits [8, 12] emphasizing the role of aMCI as a possible prodromal stage of dementia. Therefore, the amnestic subtype may represent a target patient population for the early detection of dementia-related cognitive alterations.

In the present study, using an emotional facial recognition test, which applies the signal detection and discrimination paradigm, we investigated memory abilities for facial identity and expression in persons with aMCI. We hypothesized that subjects with MCI show memory deficits for facial identity and expression when compared to healthy elderly controls. Furthermore, recent evidence suggested that recognition of negative emotions is particularly impaired in MCI [13]. Therefore, the current study used “happy” and “angry” face stimuli to test whether valence effects are present, and potentially differ between control and MCI groups.

## Patients and methods

### Patients

A total of 41 elderly control subjects (24 female; age 71±6 years) and 37 individuals with aMCI (15 female; age 75±8; all multiple-domain) were included in the present study. All subjects signed informed consent and the study was approved by the cantonal ethic committee of canton Zürich, Switzerland. The subjects were recruited from the memory clinic of the department for geriatric psychiatry in Zürich. Control subjects participated voluntarily in the study. They were tested for normal cognitive function, and matched for socioeconomic and educational background. This study was carried out in accordance with the Code of Ethics of the Worl Medical Association (Declaration of Helsinki) for experiments involving humans and was approved by the local ethic committee of the University of Zürich (Permit number: KEK-ZH-Nr.2010-0468).

The cognitive status of study participants was ascertained by clinical and laboratory examination, neuropsychological testing (CERAD-Plus with MMSE, trail making test and S-word verbal fluency task, digit span, stroop test, Kramer test, figural fluency test and clock drawing test) and neuroimaging (Magnetic Resonance Imaging; only in study participants with cognitive disturbances as a standard assessment to rule out structural brain pathology) prior to study enrollment. Common criteria for the classification of MCI were used [7]. The diagnostic criteria for MCI included a) concern regarding a change in cognition from the patient, knowledgeable informant, or from a skilled clinician observing the patient, b) objective evidence of impairment (from cognitive testing) in 1 or more cognitive domains including memory, executive function, attention, language, or visuospatial skills, c) preservation of independence in functional abilities and d) no evidence of a significant impairment in social or occupational functioning (ie. not demented) [2, 4, 7]. Impaired cognition was diagnosed by licensed clinical neuropsychologists if the performance on at least one test in that domain (memory, attention, information processing speed, language, visuospatial/constructional ability or executive function) was at least 1.5 standard deviations below age and education-matched normative means. MCI patients were classified according to the criteria of Peterson into aMCI [3]. Depression was excluded using the Hospital Anxiety and Depression Scale (cut off ≥11) [14] and Montgomery Asberg Depression Scale (cutt off ≥22) [15]. Clinical Dementia Rating and Instrumental Activities of Daily Living were other applied assessment scales. Exclusion criteria were as follows: concomitant cognitively active and sedative medication, drug abuse, critical or medically unstable illness, impairment of vision and limitation of judgement capability.

### Experimental stimuli and facial picture recognition test

Photographs of male faces with positive, negative or neutral expressions were used for the E-prime based (Psychology Software Tools Inc., Sharpsburg, PA, USA) memory test. The test procedure and the construction of experimental stimuli have been presented in detail previously [16–18]. However, pilot assessments in our cognitive lab had revealed that the PC-based test procedures, which include text readings and mandatory button pushes, confused elderly subjects, especially those with a diagnosis of MCI. Thus, a verbal response (instead of a button push) version of the test was used to assure that cognitively mildly impaired and elderly subjects inexperienced in the use of computer devices would be equally able to complete testing, and also to exclude missing responses. Participants were asked to respond promptly to verbal questions given by the experimenter (male health executive) present in the same room, however, in a speed corresponding to their individual convenience. Verbal responses were immediately coded by the experimenter, but verbal reaction time was not recorded. During the acquisition phase of the facial recognition test, a total of 52 randomly assorted different male portraits with 26 positive (happy) and 26 negative (angry) expressions were presented on a computer screen approximately 60 cm in front of seated participants. Each portrait was presented for 10 seconds. There was a 3 seconds interval between each picture. Subjects verbally indicated whether the portrait showed a happy or angry facial expression and furthermore, rated by finger pointment on a visual analogue scale (anchored 0 to 100) the actual level of confidence in their facial emotion expression discrimination. The time needed to finalise the coding of both decisions was logged, and subjected to statistical analysis. This part of the test was done to assure appropriate encoding of the faces. Memory performance was tested twice 5 and 30 minutes after the end of the encoding period. During memory testing, 52 portraits with neutral facial expressions were presented, 26 of the portraits showed individuals presented earlier during the acquisition phase (OLD: 13 formerly angry and 13 formerly happy) and 26 pictures showed new faces (NEW) never presented before. After each presentation, participants were asked to indicate whether they had seen the face previously (OLD) or not (NEW), and to additionally point by finger on a visual analogue scale (anchored 0 to 100) according to their actual level of confidence in their discriminatory decision. After that, subjects were asked to state whether the actual neutral face portrait had been presented with a negative or a positive expression during the earlier acquisition phase. Performance in this particulat part of the test was very low and close to chance level, so that reporting of these results is omitted. Furthermore, the final coding time, although logged by the PC, does not represent a valid estimate of participants’ reaction time, since the reaction times in the identity recognition and facial expression memory part may have differed. Therefore, the coding time will not be analysed, but discarded from analysis.

### Parameter calculation and statistic

Discrimination indices (d’), were derived from signal detection theory and calculated to get a bias-free measure of memory strength for identity recognition [19, 20]. Response bias (C) estimation was based on published standards [21]. In brief, hit rates and false alarm rates per time point were calculated for each subject by taking the relevant number of hits and false alarms and dividing this number by the corresponding number (n) of old/new pictures (n = 13 for the two expression specific hits / n = 26 for total hits / n = 26 for false alarms). The indices d’ and C were calculated as follows:

d’=Z(hitrate)‑Z(falsealarmrate)

$$C=\left(Z{\left(hit\;rate\right)}^{2}+Z{\left(false\;alarm\;rate\right)}^{2}\right)/2$$

Since inverse Z-transformation of hit and false alarm rates of 0 or 1 is not possible these values were corrected by adding ½ n during the calculation of false alarm and hit rates of 0 and subtracting ½ n during the calculation of false alarm and hit rates of 1, with n equal to the total number of old and new portraits, respectively.

### Sample analysis

T-Test were used to test for GROUP differences drung the acqusition phase. Data are presented in Table 1.

**Table 1:** Acquisition of emotional faces.

	Control			MCI			Group t-test	
	Mean	N	(SEM)	Mean	N	(SEM)	T	*p*
HR-Angry	0.933	41	(0.011)	0.933	37	(0.010)	-0.05	.*96*
HR-Angry	0.933	41	(0.011)	0.933	37	(0.010)	-0.05	.*96*
HR-Happy	0.954	41	(0.010)	0.960	37	(0.007)	-0.42	.*68*
SR H-Angry	53.20	41	(1.452)	48.63	37	(1.471)	2.20	**.*03***
SR F-Angry	22.31	28	(1.769)	24.06	27	(2.712)	-0.55	.*58*
SR H-Happy	50.23	41	(1.436)	47.98	37	(1.451)	1.10	.*27*
SR F-Happy	20.31	18	(2.640)	26.25	18	(2.509)	-1.63	.*11*
ZR H-Angry	0.150	41	(0.037)	0.070	37	(0.038)	1.49	.*14*
ZR F-Angry	-1.436	28	(0.097)	-1.456	27	(0.187)	0.10	.*92*
ZR H-Happy	-0.013	41	(0.040)	0.056	37	(0.042)	-1.19	.*24*
ZR F-Happy	-1.539	18	(0.148)	-1.227	18	(0.117)	-1.65	.*11*
TI H-Angry	5224	41	(403)	4779	37	(321)	0.85	.*39*
TI F-Angry	6641	28	(683)	8706	27	(125)	-1.46	.*15*
TI H-Happy	4695	41	(179)	4384	37	(274)	0.96	.*34*
TI F-Happy	7226	18	(655)	5900	18	(777)	1.30	.*20*

**Hit rate (HR) of correctly discriminated Happy vs. Angry faces.** False alarm (FA) rates may be calculated (e.g. FA-Angry = 100 minus HR-Happy). Confidence ratings (SR), z-transformed SR (ZR), and response time (TI) of discriminatory decisions per hit (H) and false (F) alarm. Note the varying n, reflecting that FA were present only in a subgroups of participants. Data represent mean +/- SEM, italic p-values, **bold** when statistically significant.

Mixed design ANOVAs suitable for repeated measures were constructed with GROUP (levels: aMCI, Control) as the only between-subject factor, and TIME (levels: 5, 30 min) and VALENCE (levels: angry, happy facial expression) as within-subject factors. Data are presented in Table 2

**Table 2:** Recognition memory testing for face identity with respect to valence effects.

	Control			MCI	ANOVA F[1:76]						
	5 Min	30 Min	5 Min	30 Min	Group	Val	GxV	Time	TxG	VxT	VxTxG
d’ Hap	0.94 (0.09)	0.62 (0.09)	0.66 (0.08)	0.35 (0.09)	10.5	22.8	0.1	12.5	0.0	4.6	0.0
d’ Ang	0.64 (0.08)	0.52 (0.08)	0.41 (0.07)	0.26 (0.08)	**.*002***	**.*0001***	.*71*	**.*0001***	.*96*	**.*04***	.*85*
C Hap	0.44 (0.07)	0.29 (0.04)	0.51 (0.08)	0.56 (0.11)	4.1	9.3	0.0	0.0	2.4	2.3	0.0
C Ang	0.32 (0.06)	0.28 (0.04)	0.40 (0.06)	0.53 (0.12)	**.*04***	**.*003***	.*87*	.*98*	.*12*	.*13*	.*84*
HR Hap	0.70 (0.02)	0.61 (0.02)	0.64 (0.04)	0.59 (0.04)	0.6	21.9	0.3	4.1	0.8	4.7	0.1
HR Ang	0.60 (0.02)	0.57 (0.03)	0.56 (0.04)	0.57 (0.04)	.*43*	**.*0001***	.*61*	**.*05***	.*38*	**.*03***	.*79*
SR Hap	72.6 (2.1)	70.9 (2.4)	70.0 (2.4)	71.6 (2.2)	0.1	3.6	0.7	4.5	2.3	2.9	3.9
SR Ang	69.1 (2.3)	71.6 (2.3)	68.6 (2.2)	70.9 (2.2)	.*73*	.*06*	.*42*	**.*04***	.*13*	.*09*	.*06*

**Sensitivity of OLD/NEW facial identity discrimination (d’) per facial valence at acquisition, response bias (C), hit rate (HR), and confidence ratings (SR).** Data of the left part of the Table represent mean (SEM) per Group, Valence (Val, V), and Time (T: 5 vs 30 Minutes). Data of the right part of the Table represent ANOVA statistics, F-values, and *italic* p-values, ***bold*** when statistically significant, per factor and interaction.

Mixed design ANOVAs suitable for repeated measures were constructed with GROUP (levels: aMCI, Control) as the only between-subject factor, and TIME (levels: 5, 30 min) as the only within-subject factors for False alarm related parameters. Data are presented in Table 3.

**Table 3:** False alarms.

	Control		MCI		ANOVA F[1:76]					
	5 Min.	30 Min.	5 Min	30 Min	Group	p	Time	p	GxT	p
FR	0.37 (0.02)	0.39 (0.03)	0.42 (0.03)	0.48 (0.04)	3.9	**.*05***	6.2	**.*01***	1.4	.*24*
SR FA	66.1 (2.2)	68.8 (2.3)	65.6 (2.6)	69.0 (2.0)	**0.0**	.*94*	7.3	**.*01***	0.1	.*74*

**False alarm rate (FR), and confidence ratings (SR) of false alarms.** Data of the left part of the Table represent mean (SEM) per Group, and Time (T: 5 vs 30 Minutes). Data of the right part of the Table represent ANOVA statistics, F-values, and *italic* p-values, ***bold*** when statistically significant, per factor and interaction.

Interaction effects involving GROUP were a-priori planned to further analisis by two-sided paired t-tests. The association of hit (HR) and fals alarm rates (FR) was analysed (after inverse Z-transformation) by Pearson correlation, and simple regression analyses. Homogeneity of slopes between MCI and control groups was tested by a general linear model (GLM). All statistical analyses were performed with SAS (V 9.1.3) on a Win7 (64 bit) platform. An alpha = 0.05 level was considered to reflect statistical significance. All data provided reflect mean +/- SEM, unless otherwise stated.

There was a significant difference in age between aMCI and control subjects (t = 3.0, p = 0.005). Therefore, within each group it was tested whether age was correlated with indices of memory performance in order to decide whether to include it as a covariate in subsequent analyses. However, age was not significantly correlated with memory performance in neither the control subjects, nor the aMCI group. Furthermore, excluding the youngest subjects from the CS group did not significantly change the results. Thus, age was not introduced as a covariate.

## Results

### Acquisition phase and analyses of differences between MCI and control subjects

All data sampled during the acquisition phase is provided in Table 1. Comparison of discriminatory performance (discriminating Happy from Angry, and vice versa) revealed no differences between groups. Hit rates were high (approx. 95%). There was a significant difference in confidence ratings. Control subjects were more confident in their decision on true Angry faces than MCI patients. However, z-transformed confidence ratings did not further substantiate this small difference. No other difference was statistically different, indicating that acquisition of test stimuli was rather similar between groups.

### Recognition memory for faces

A mixed design ANOVA with the dependent variable “sensitivity index” d’ (see Table 1) revealed a significant GROUP effect (higher d’ in the control group), a significant VALENCE effect (higher d’ for happy encoded faces), a significant TIME effect (lower d’ after 30 min than after 5 min), and a significant VALENCE X TIME interaction. No interaction involving group reached statistical significance. The significant VALENCE X TIME interaction may likely depent on ceiling (or initial) values effects, and was not further explored, since it did not involve Group.

A mixed design ANOVA with the dependent variable ‘response bias’ C (see Table 2) revealed a significant GROUP effect (higher C in the MCI group), and a significant VALENCE effect (higher C for Happy encoded faces), but no other main or interaction effects.

A mixed design ANOVA with the dependent variable ‘hit rate’ (see Table 2) revealed a significant Valence effect (higher hit rate for Happy encoded faces), but no other main or interaction effects involving Group. A similar result pattern was found for confidence ratings.

A mixed design ANOVA with the dependent variable ‘false alarm rate’ (see Table 3) revealed a significant GROUP effect (higher false alarms in the MCI group), a significant TIME effect (higher flase alarms after 30 min than after 5 min), but no interaction involving GROUP. This was also true for the confidence ratings related to false alarm decisions.

**Fig 1:**
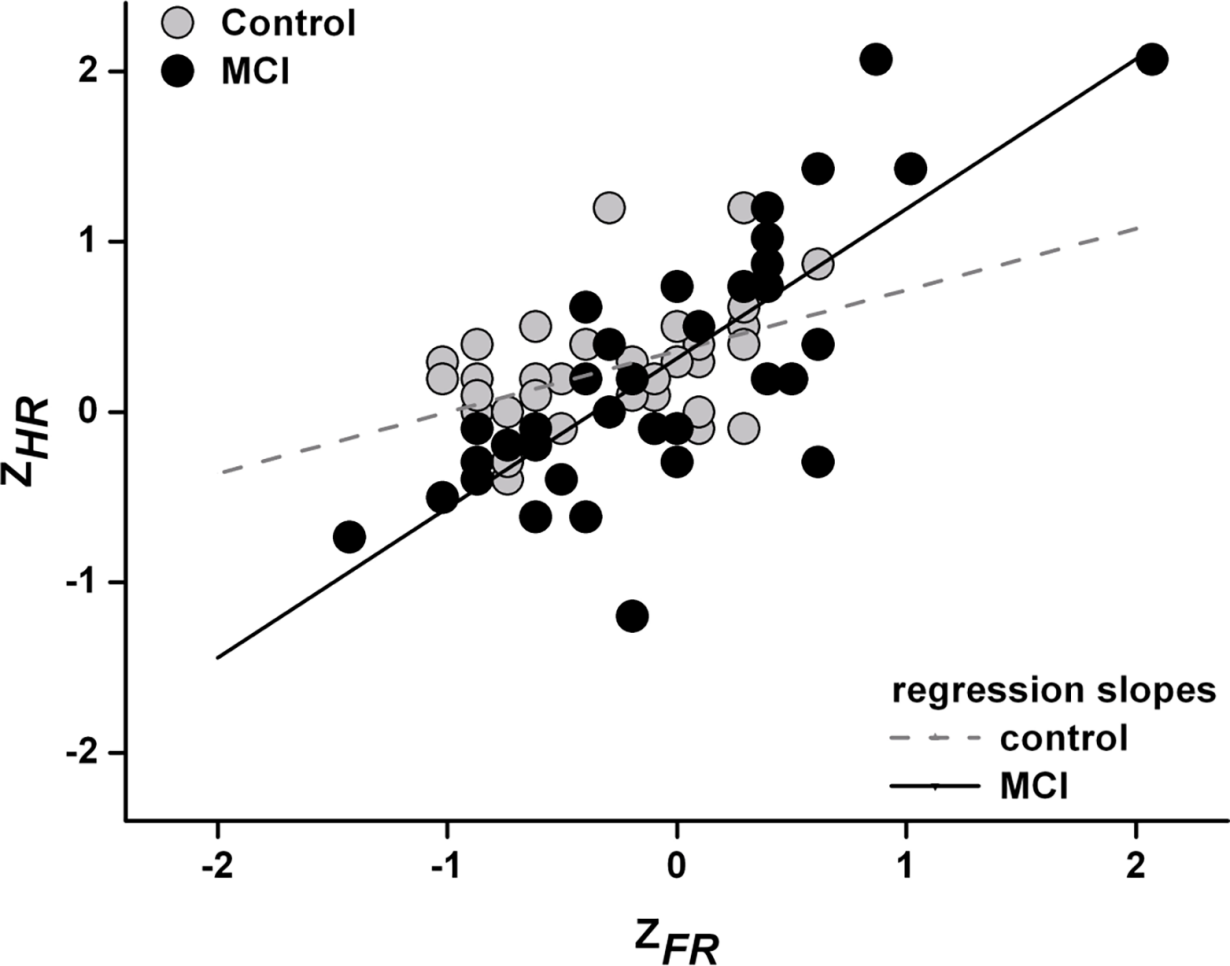
Inverse Z-transformed hit (Z_HR_) and false alarm rates (Z_FR_) per group (control = grey, MCI = black circles) and respective regression lines.

The association of hit and false alarm rates (inverse Z-transformed) were tested per group by Pearson correlation analysis and revealed r = 0.46 (p = .003) for the control group, and r = 0.78 (p < .0001) for the MCI group. Regression equations were as follows (control: Z_HR_ = 0.358 + Z_FR_ * 0.359 (SEM = 0.110, t = 3.2, p = .003 / MCI: Z_HR_ = 0.315 + Z_FR_ * 0.878 (SEM = 0.120, t = 7.3, p < .0001), and significantly different between groups (F[1:76] = 8.9, p = .004) (see also Fig 1).

## Discussion

Elderly aMCI subjects and healthy controls were studied by a cued facial identity recognition memory task. Memory performance (d’) was lower in aMCI as compared to control subjects, response bias (C) was higher in aMCI, as was false alarm rate (false indication of NEW faces as OLD). No other effects involving group emerged. Especially, hit rate (correct indication of OLD faces as OLD) was similar between aMCI and control subjects.

It is very unlikely that these results stem from attentional differences during acquision. The task during acquisition was to discriminate happy from angry faces, and there was apparently no major difference in performance measures (correct response rate, confidence ratings, response times) between groups. Interestingly, the liberalization of the response bias in aMCI patients favors false alarm rate as well as hit rate. Hit rate is a direct measure of the percentage of correctly indicated OLD faces. Certainly, this index is not an appropriate neuropsychological estimate of memory function, since it is not corrected for the response bias, and thus, does not take into account the potential expenses induced by false alarms. However, from a social perspective the hit rate may be the most important variable, since it supports appropriate reactions to “old” peers at the (less relevant) expense of wrong familiarity judgements of “new” (and likely never see again) strangers. Thus, the increased response bias in aMCI subjects may reflect an implicit, socially motivated compensatory mechanism maintaining an almost similar hit rate of OLD faces in aMCI patients as compared to age-matched healthy controls. This interpretation is supported by regression analysis indicating that the regression line between false alarm rate and hit rate is steeper in aMCI patients as compared to control subjects, thus, their hit rate profits more from an increase in false alarm rate. However, we want to emphasize that our interpretation of the results as a socially motivated compensatory mechanisms is only a speculation. Further studies are necessary to control for non-social memory performance, especially for false alarm rates regarding non-social stimuli.

In both groups memory performance was always better for faces presented with a happy vs. angry facial expression during acquisition. Early studies demonstrated that neutral faces are better recognized when they had been previously encountered with happy rather than angry expressions [22]. Also in previous findings, healthy elderly subjects showed a positive bias towards positive facial identities in facial recognition task [16, 23]. The ability to recognize both facial identity and emotional expression declines with advanced age in healthy individuals but positive facial expressions are better recognized in all age groups. On the other hand, this positive bias is absent in elderly depressed patients [17]. Identity recognition memory performance for both positive and negative stimuli, but more for positive stimuli, decreases in depression similar to the findings for patients with aMCI. Depression has a significant influence on the recognition of overall emotions and leads to a negative bias [11]. The present study did not find any interaction of group and valance, and thus, does not suggest that this bias may represent a differentiating factor between aMCI and depression [13]. This is in accordance with experiments demonstrating a relatively preserved recognition bias for positive faces in patients with aMCI [23].

Recent research has found that deficits in visual recognition of facial emotions in aMCI individuals may arise already in the earliest stage of memorization, during the encoding of facial emotions [24]. Electrophysiological evidence also points to impairments in face recognition processes and emotional memory in the early coding stages in aMCI [25]. Voxel-based morphometry revealed some association between these deficits in aMCI and atrophy in frontal and occipito-temporal regions [24]. Since the disorder was not related to the extent (single or multiple domain) of the cognitive impairment but rather to the involvement of frontal brain networks, the authors concluded that in prodromal stages of dementia, frontal symptoms may represent a significant signal of emotional recognition disorder [24]. aMCI subjects with frontal symptoms and atrophy may present early disorders in the visual perception of negative facial expressions, irrespective of their general cognitive decline. Moreover, cognitive aspects of empathy may be reduced in aMCI and the general deficit in visual form discrimination is associated with temporo-mesial and occipital atrophy [24].

In accordance with the present results facial recognition was found to be primarily impaired in the amnestic subtype [10, 11]. In a study using the Penn Emotion Recognition Test and comparing aMCI single domain (MCIs) and aMCI multiple domains (MCIm) subjects with control subjects and AD patients, patients with MCIm and AD were impaired in the recognition of overall emotions (sad, fearful, and neutral faces) whereas MCIs and control subjects did not differ significantly in their memory performance [11]. The deficits increased with the severity of AD. The authors concluded that the MCIm subtype may represent a preliminary stage of AD with diminished recognition capacity for sad, fearful, and neutral faces. In the other study assessing MCIs and MCIm subjects with the Florida Affect Battery, the memory performance was intact in the MCIs group and significantly impaired in the MCIm group [10]. In addition, the performance on facial affect discrimination in the MCIm group correlated robustly with performance on tests of frontal/executive function. The authors assumed that the poorer performance of the MCIm group may be attributed to more advanced degeneration in specific regions of the brain, especially in medial temporal lobe structures.

The emotional recognition difficulties in aMCI may have some implications for the social interactions of these individuals. In fact, impaired facial emotional expression detection was found to be associated with a social behavior disorder that affects social life in early AD [26]. In the same study, the authors were unable to find any expression recognition deficits in aMCI. Since only 10 individuals with aMCI were included in the study, the authors themselves mentioned that the results have to be interpreted with caution because of the small sample size. Although the patients with aMCI seem to not benefit from fearful emotional content which facilitates stimulus recognition (emotional enhancement effect) in healthy controls [24], there is some evidence that emotional messages can improve episodic memory in aMCI [25, 27–29]. Taking the early deficits in emotion recognition in aMCI into account, emotional stimuli, especially the positive ones, may help to improve cognition in aMCI, since, although with a reduced performance, individuals with aMCI still recognize positive facial identity better than negative pictures.

## Conclusion

Memory for facial identity recognition was impaired in patients with MCI and these subjects showed a liberal response bias toward OLD ascription of NEW items. The identity recognition of happy faces was favored both in CS and MCI. Facial identity memory deficits may contribute to difficulties in social behavior and interpersonal interactions in MCI.

## Supporting information

S1 Table Participant characteristics. Excel sheet including group, sex, age, BMI, and signal detection variable d-prime (d’) of identity recognition, memory bias (C), raw probability (p) of hits and false alarms, as well as confidence ratings (r) of “happy” and “angry” faces (whenever appropriate) for all individuals participating in the study.
(TIF)
