# Supplementary figures and images for: Memory deficits for facial identity in patients with amnestic mild cognitive impairment (MCI)
Source: PLoS One. 2018 Apr 19;13(4):e0195693. doi: 10.1371/journal.pone.0195693 (PMC5908082; doi:10.1371/journal.pone.0195693)

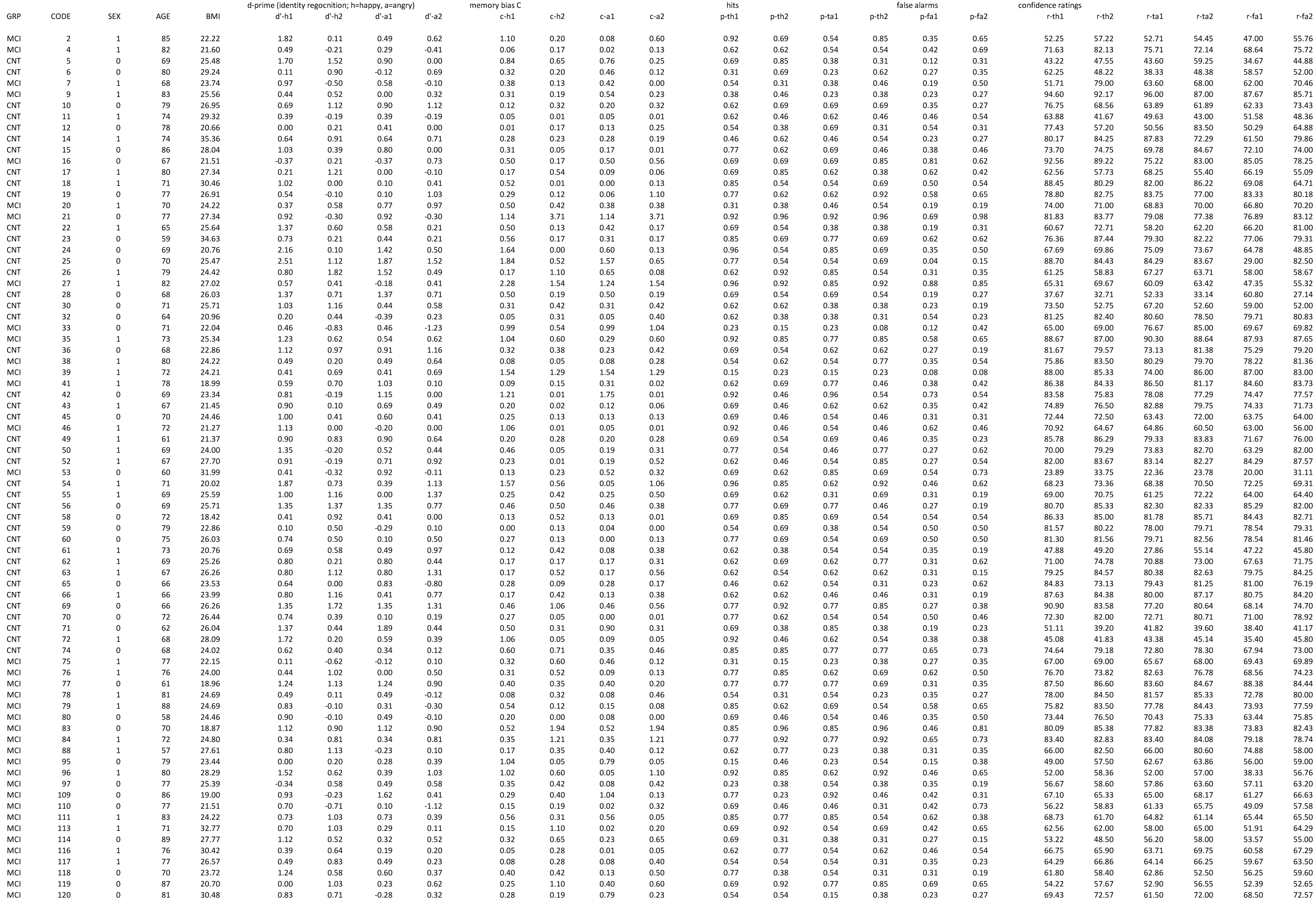

Supplement: S1 Table — Excel sheet including group, sex, age, BMI, and signal detection variable d-prime (d’) of identity recognition, memory bias (C), raw probability (p) of hits and false alarms, as well as confidence ratings (r) of “happy” and “angry” faces (whenever appropriate) for all individuals participating in the study. (TIF) [file pone.0195693.s001.tif]
